# Dr. Jarvis S. Wight

**Published:** 1901-12

**Authors:** 


Dr. Jarvis S. Wight, of Brooklyn, died at his home November 17, 1901, at the age of 67 years. He was born in Centerville, Allegany County, N.Y., in 1834, and was a descendant of Thomas Wight, who emigrated from the Isle of Wight to America in 1635. After being graduated from Tufts College in 1861, he studied at the College of Physicians and Surgeons, New York, and at the Long Island College Hospital, where he received his degree in 1864. For a year he served as an assistant surgeon to the volunteers in the civil war, and at its close became dispensing surgeon to the Long Island College Hospital, lecturer on skin diseases, professor of materia medica and principles and practice of surgery, and professor of operative and clinical surgery, a position which he held up to the time of his death. For many years he was the registrar of the college. He was consulting surgeon at St. Mary's Hospital, and at the Eastern District Hospital. Dr. Wight was a member of several local, state, and national medical societies.

